# Insufficient microwave ablation-induced promotion of distant metastasis is suppressed by β-catenin pathway inhibition in breast cancer

**DOI:** 10.18632/oncotarget.22859

**Published:** 2017-12-01

**Authors:** Peng Kong, Hong Pan, Muxin Yu, Lie Chen, Han Ge, Jin Zhu, Ge Ma, Li Li, Qiang Ding, Wenbin Zhou, Shui Wang

**Affiliations:** ^1^ Department of Breast Surgery, The First Affiliated Hospital with Nanjing Medical University, 210029 Nanjing, China

**Keywords:** microwave ablation, breast cancer, epithelial-mesenchymal transition (EMT), β-catenin, metastasis

## Abstract

Microwave ablation (MWA), a thermal ablation, is an effective treatment for breast cancer. However, residual breast cancer is still detected. The biological characteristics of residual breast cancer after thermal ablation remain unknown. To mimic insufficient MWA *in vitro*, breast cancer cells were treated at 37°C, 42°C, 45°C, 47°C and 50°C for 10 mins, the 37°C as control group. Insufficient MWA induced EMT-like changes of residual breast cancer by down-regulation of E-cadherin and up-regulation of vimentin and N-cadherin *in vitro* and *in vivo*. For the first time, we reported insufficient MWA promoted distant metastasis of residual breast cancer *in vivo*. Reduced β-catenin expression by siRNA diminished the EMT-like phenotype and enhanced migration capability induced by heat treatment in breast cancer cells. Moreover, ICG001, a special inhibitor of β-catenin pathway, depressed EMT of residual tumor and distant metastasis in an insufficient MWA nude mice model of breast cancer. In conclusion, our results demonstrate that insufficient MWA promotes EMT of residual breast cancer by activating β-catenin signal pathway, resulting in enhanced distant metastasis of residual breast cancer. In addition, the effectiveness of ICG001 in suppressing enhanced metastasis of residual breast cancer is preliminarily validated.

## INTRODUCTION

Microwave ablation (MWA), a minimally invasive therapy, presents the advantages of higher ablation temperature, larger ablation range and shorter ablation time compared with other thermal ablations such as radiofrequency ablation (RFA), high-intensity focused ultrasound and laser ablation in the treatment of solid tumors [1–3]. The efficiency and security of MWA in the treatment of breast cancer have been confirmed in our previous studies [4–6]; however, residual tumor has been found in several patients and animal models after MWA. To reduce the residual tumor, MWA combined with chemotherapy or ^131^I radiation therapy have been investigated, and the results have shown that combined therapy significantly improved the ablation volume and the survival time, but the residual tumor still exists [7, 8]. Residual tumor and local recurrence after thermal ablation are also the great challenge of these minimally invasive treatments. In hepatocelluar carcinoma (HCC) [9–11], the local recurrence after RFA shows a more malignant phenotype with raised tumor growth and distant metastasis. To the best of our knowledge, there have been no reports on biological characteristics changes of residual breast cancer after thermal ablation.

Epithelial-mesenchymal transition (EMT) is involved in various physiological and pathological processes [12]. EMT has been confirmed to accelerating the progression by inducing distant metastasis [13–15]. Several cell signaling pathways, such as transform growth factor β (TGF-β), Wnt, integrin and Notch, have been shown to induce EMT by activating transcription factors Snail, Slug, Twist and Zeb1/2 in breast cancer [16–19]. In particular, the Wnt/β-catenin pathway plays an important role in tumor initiation and prognosis in breast cancer [20]. β-catenin, the concernment molecules in Wnt pathway, acts as a regulator of transcription in the nucleus. It has been reported that over 60% breast cancers with high expression levels of β-catenin [21], which is correlated with poor prognosis.

Residual tumors after thermal ablation are treated with sublethal heat. To mimic insufficient MWA, breast cancer cells treated with different temperatures were applied to determine biological characteristics *in vitro*. Here, biological characteristics changes of residual breast cancer after MWA and the underlying mechanism were reported *in vitro* and *in vivo*. We found the enhanced metastatic capacity of residual breast cancer was related to β-catenin pathway. The strategy to depress the enhanced metastatic capacity was also reported.

## RESULTS

### Heat treatment decreased survive rate and raised apoptosis in breast cancer cells *in vitro*

**Figure 1:**
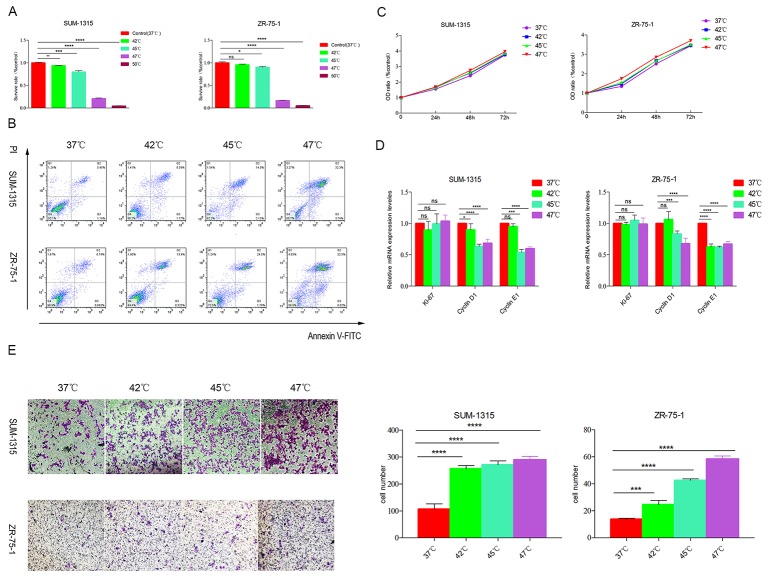
Heat treatment promoted the migration but not proliferation of survived breast cancer *in vitro* **(A)** SUM-1315 and ZR-75-1 cells were exposed to 42°C, 45°C, 47°C and 50°C for 10min, and the survive rate of heated cells were tested after 24h by CCK8 assay. The cell survival rate decreased with the increasing temperature. **(B)** The apoptosis rates tested by flow cytometry after 48h of heated cells were significantly higher than that in control group. **(C)** At 72h after heat treatment, the proliferation of survived cells was tested. Heat treatment did not promote the proliferation of survived SUM-1315 and ZR-75-1 cells. **(D)** The mRNA level of Ki-67 was not enhanced after heat treatment. **(E)** The survived cells showed enhanced migration compared with control group at 72h after heat treatment. ^*^p<0.05,^**^p<0.01, ^***^p<0.001,^****^p<0.0001.

To mimic insufficient MWA *in vitro*, SUM-1315, ZR-75-1 breast cancer cells were treated at 37°C, 42°C, 45°C, 47°C and 50°Cfor 10mins, the 37°C as the control group. After 48h, the survive rates were tested by CCK8 assay. The cell survival rate decreased with the increasing temperature. When treated with 42°C, about 93.91% SUM-1315 and 95.68% ZR-751 cells survived, whereas the survive rate decreased to 79.66% and 89.93% at 45°C. At 47°C, 20.79% SUM-1315 and 16.32% ZR-75-1 cells survived, and almost all the cells died after exposed to 50°C (Figure 1A). In apoptosis assay by flow cytometric analysis, higher apoptosis rates were observed in SUM-1315 (10.36%, 16.13%, 36.04% *vs*6.65%) and ZR-75-1 (13.63%, 26.28%, 38.52%*vs*7.41%) breast cancer cells after treated with 42°C, 45°C and 47°Cthan that in control group *in vitro* (Figure 1B).

### Enhanced migration but not proliferation of survived breast cancer cells after heat treatment *in vitro*

After heat treatment, the proliferation and migration of survived cells were determined. The proliferation rates at 24h, 48h and 72h of heat-treated SUM-1315 and ZR-75-1 cells were the same as that in control group (Figure 1C). Moreover, no significant difference of Ki-67 (a proliferation-related transcription factor) was observed among different treated groups in the two cell lines (Figure 1D).

SUM-1315 and ZR-75-1 breast cancer cells were treated at 42°C, 45°C and 47°C for 10min, and the migration capacity of survived breast cancer cells were investigated after 3 days. All heat-treated SUM-1315 breast cancer cells exhibited higher migration than that in the control group (256.2±11.72, 270.8±14.44, 289.5±12.68 *vs* 106.6±19.28). The ZR-75-1 cells treated with 42°C, 45°C and 47°C also showed higher migration than that in control group (24.50±3.04, 42.50±1.07, 58.30±2.35 *vs* 13.70±0.70, Figure 1E). All the results suggested that heat-treated breast cancer cells showed higher migration ability.

### Heat treatment induced EMT-like morphological and characteristic changes in breast cancer cells

**Figure 2:**
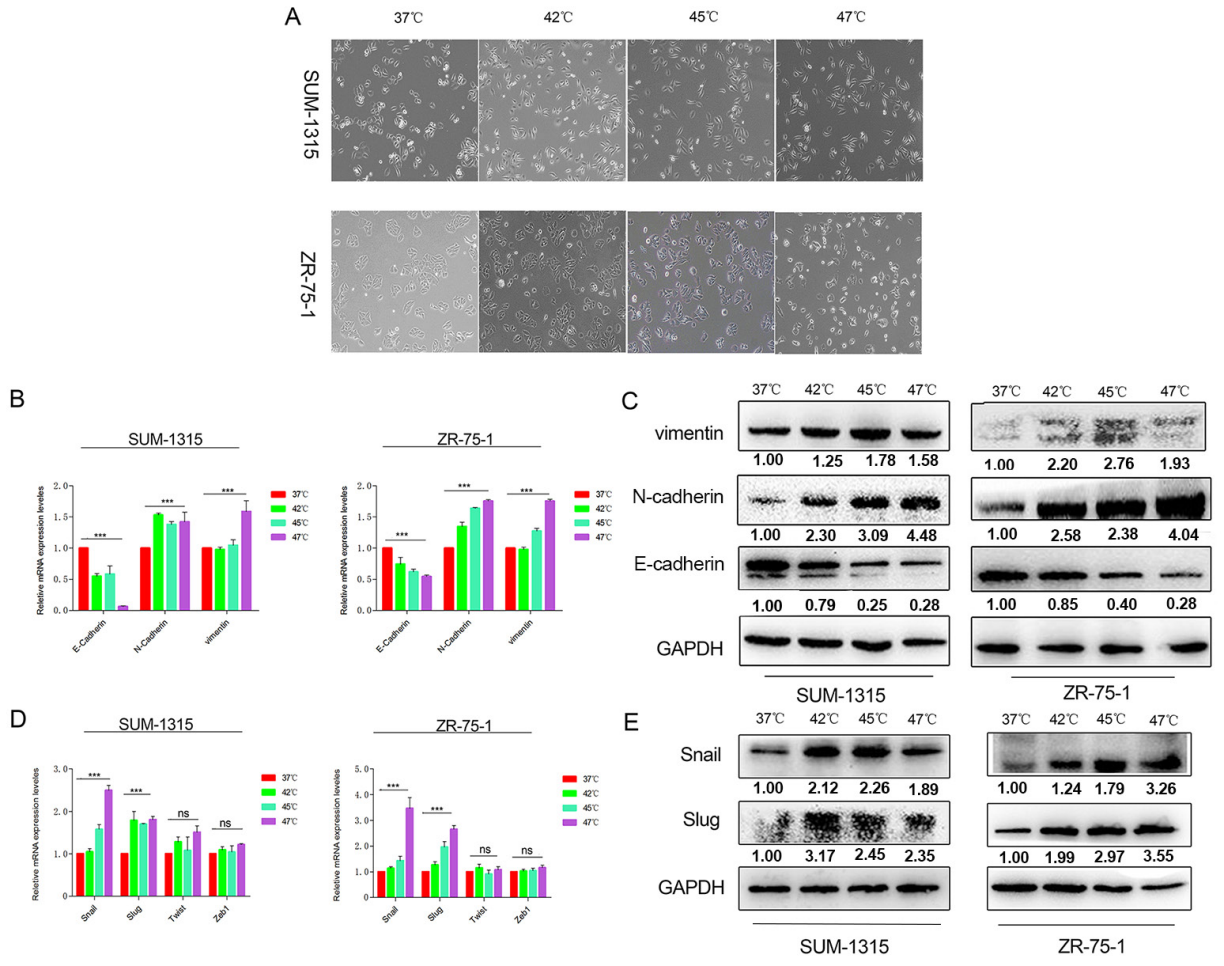
Heat treatment induced EMT in SUM-1315 and ZR-75-1 cells **(A)** The obvious spindle shape morphological other than typical epithelial cobblestone appearance of heat-treated breast cancer cells was observed at 48h after heat treatment. RT-PCR **(B)** and western blot **(C)** showed down-regulation of E-cadherin and up-regulation ofN-cadherin and vimentin in breast cancer cells after heat treatment. Raised expressions of EMT transcripts Snail and Slug after heat treatment were investigated by RT-PCR **(D)** and western blot **(E)**. ^***^p<0.001.

The cell morphological of SUM-1315 and ZR-75-1 breast cancer cells gained EMT-like morphological at 48h after heat treatment. The obvious spindle shape morphological other than typical epithelial cobblestone appearance of heat-treated breast cancer cells was observed (Figure 2A). To confirm the EMT changes in heat-treated breast cancer cells, EMT-related cell markers (E-cadherin, N-cadherin and vimentin) and transcripts (Snail, Slug, Twist and ZEB1) were analyzed by western blot and reverse-transcriptase polymerase chain reaction (RT-PCR). The EMT marks detected by western blot showed a reduced expression of epithelial cell marker E-cadherin, and the raised expression of the mesenchymal cell markers (N-cadherin and vimentin) (Figure 2C) in survived cells. Furthermore, raised expressions of EMT transcripts (Snail, Slug) were investigated by western blot (Figure 2E). In addition, the mRNA levels of E-cadherin, N-cadherin, vimentin, Snail and Slug in heat-treated cells detected by RT-PCR showed the similar trends as protein levels (Figure 2B, 2D).

### Insufficient MWA reduced primary tumor burden but promoted distant metastasis in breast cancer xenografts

**Figure 3:**
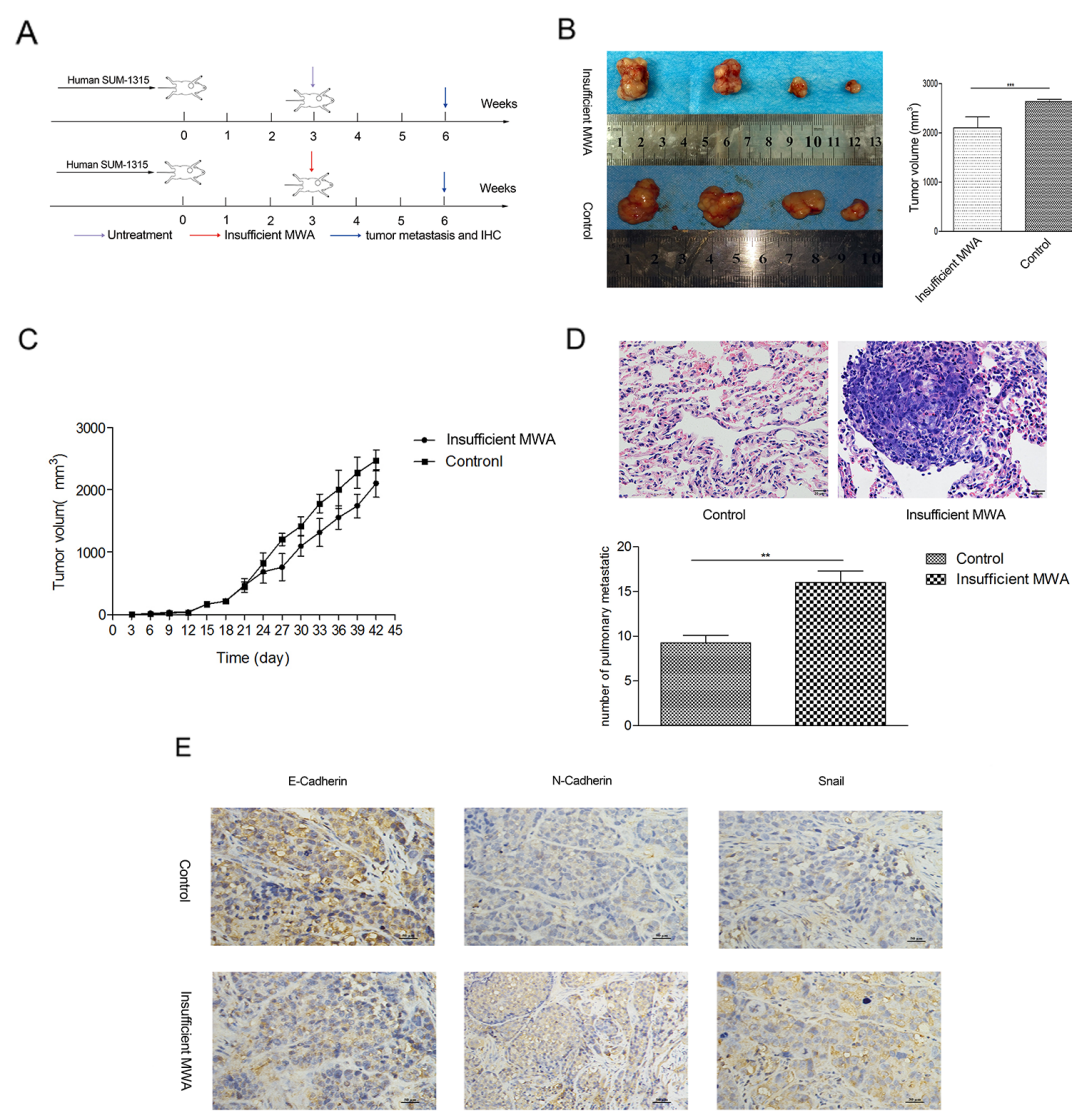
Insufficient MWA reduced primary tumor burden but promote distant metastasis in breast cancer xenografts **(A)** Experiment design of the *in vivo* test. **(B)** In insufficient MWA group, the tumor volume was evidently smaller than that in control group (2104±223.1 *vs*2431±211.3, ^***^p<0.001). **(C)** Tumor growth curve indicated tumor volume decreased after insufficient MWA, while the tumor growth rate was the same as control group. **(D)** Representative images of HE staining of pulmonary metastasis foci, and insufficient MWA induced more pulmonary metastasis in SUM-1315 nude model. **(E)** Immunohistochemical displayed down-expression of E-cadherin in the tumor tissue in insufficient MWA group, and over-expression of N-cadherin and Snail also detected.^**^p<0.01, ^***^p<0.001.

To evaluate the insufficient MWA on residual tumor malignancy, we utilized orthotopic nude mouse SUM-1315 breast cancer model (Figure 3A). In insufficient MWA group, the tumor volume was evidently smaller than that in control group (2104±223.1 *vs*2431±211.3, *p*<0.05, Figure 3B), while the tumor growth rates were the same between two groups (Figure 3C). Immunohistochemistry (IHC) analysis further demonstrated that the residual cancer following MWA gained EMT like changes, with down-regulation of E-cadherin and up-regulation of N-cadherin and Snail in the xenograft tumors (Figure 3E). The metastatic potential of residual tumor of SUM-1315 after insufficient MWA was tested by serial lung paraffin sections. The number of pulmonary metastatic foci in the insufficient MWA group was significantly higher than that in the untreated control groups (16±1.29 *vs* 9.25±0.85, *p*<0.01, Figure 3D).

### β-catenin expression increased in heat-treated breast cancer cells *in vitro* and residual tumor after insufficient MWA *in vivo*

We detected the common EMT related proteins (TGF-β1, Notch1, β-catenin) in the pre-test by western blot (data not shown), and found the expression of β-catenin significantly increased. β-catenin is an important signaling pathways to regulate EMT. RT-PCR indicated that heat treatment (42°C, 45°C and 47°C) enhanced β-catenin mRNA level 1.5-, 2.2- and 2.3-fold at 3days in SUM-1315 cells, respectively (data not shown). In ZR-75-1 breast cancer cell, only 47°C and 45°C heat-treated subtype showed significantly higher β-catenin level, while this level was not significantly promoted in 42°Cheated cells. Therefore, 45°Cwas selected for subsequent experiments as heat-treated group.

**Figure 4:**
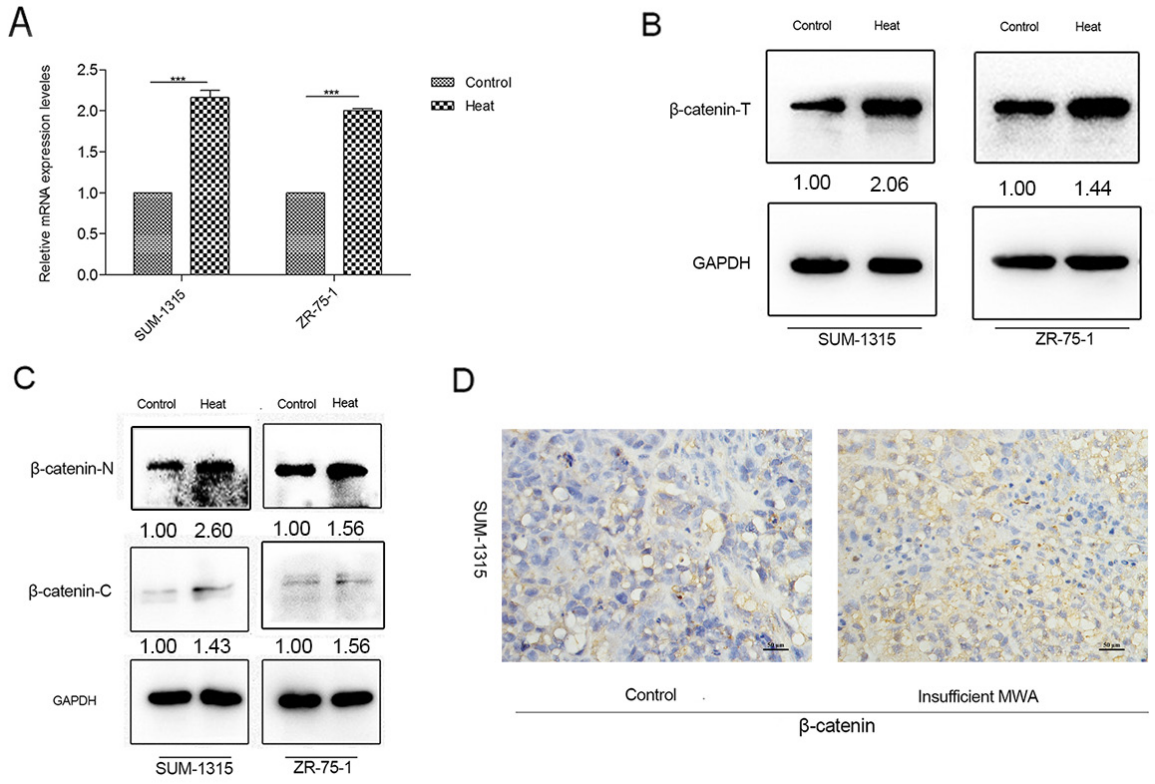
β-catenin increased in heat-treated breast cancer cells and insufficient MWA tumor tissues **(A)** Enhanced β-catenin transcription levels in SUM-1315 and ZR-75-1 cells after heat treatment were confirmed by RT-PCR. **(B)** Up-regulation of total β-catenin protein was detected by western blot in heat-treated SUM-1315 and ZR-75-1 cells. **(C)** Western blot indicated both cytoplasm and nucleus β-catenin increased in the heat-treated cells. **(D)** Immunohistochemical staining approved a raised intracellular β-catenin after insufficient MWA in SUM-1315 nude breast cancer model.^***^p<0.001.

The β-catenin mRNA level and total protein expression in heat-treated SUM-1315 and ZR-75-1 cells were significantly higher than those in control groups (Figure 4A and 4B). Moreover, western blot indicated both the expression of cytoplasm and nucleus β-catenin protein increased in the heat-treated two cell lines (Figure 4C). IHC approved a raised intracellular β-catenin after insufficient MWA in SUM-1315 nude breast cancer model (Figure 4D).

### Silencing β-catenin by CTNNB1 siRNA reduced migration ability and impeded EMT induced by heat treatment in SUM-1315 and ZR-75-1 cells

**Figure 5:**
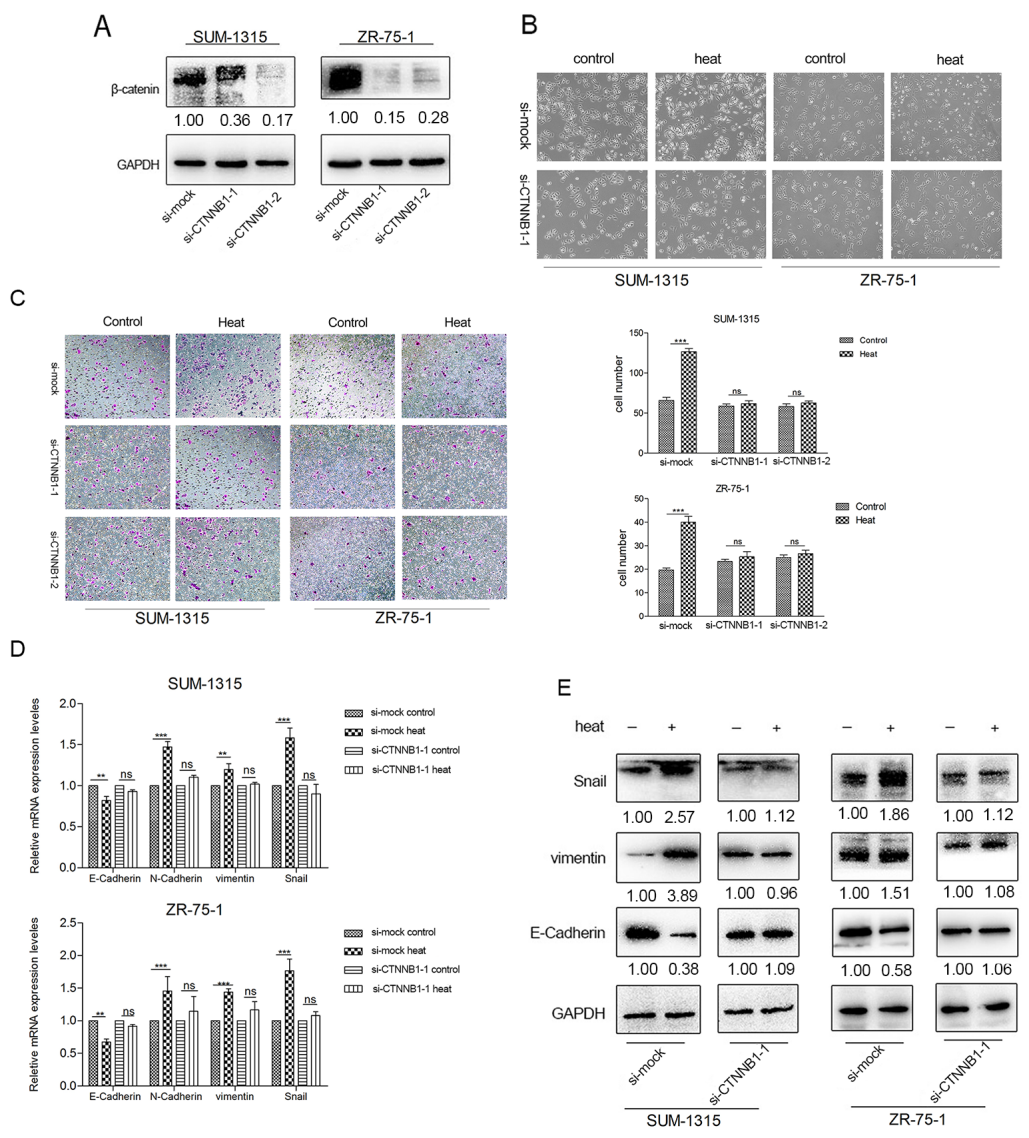
The heat treatment induced EMT in SUM-1315 and ZR-75-1 cells were inhibited by CTNNB1 siRNA **(A)** The expression of β-catenin in SUM-1315 and ZR-75-1 cells was notably reduced after silencing CTNNB1 by siRNA. **(B)** The EMT-like morphology induced by heat treatment was partially impeded by CTNNB1 siRNA. **(C)** After transfected with CTNNB1 siRNA in SUM-1315 and ZR-75-1 breast cancer cells, the enhanced migration ability induced by heat treatment was diminished. RT-PCR **(D)** and western blot **(E)** showed the EMT related markers (N-cadherin) and transcripts (Snail) were not promoted in heated breast cancer cells transfected with CTNNB1siRNA, meanwhile the down-regulation of E-cadherin was also diminished.^**^p<0.01, ^***^p<0.001.

To investigate the role of β-catenin in enhanced migration and EMT in SUM-1315 and ZR-75-1cells after heat treatment, CTNNB1 siRNA was used. After transfected with CTNNB1 siRNA, the total β-catenin protein contents in SUM-1315-siCTNNB1 and ZR-75-1-siCTNNB1 were notably less than that in SUM-1315-simock and ZR-75-1-simock cells (Figure 5A). After 45°Cheat treatment, the cell morphology of the SUM-1315-siCTNNB1 cells was the same as non-heat-treatment SUM-1315-siCTNNB1 cells; however, distinctly spindle-like changes were presented in heated SUM-1315-simock cells (Figure 5B). In migration assay, no significant differences were found in the migration cell numbers between the non-heated and heat-treated SUM-1315-siCTNNB1 cells (Figure 5C). After heat treatment, the migration cell number of the SUM-1315-simock cells was obviously higher than that of non-heat-treated SUM-1315-siMock cells (Figure 5C). The same results were observed in ZR-75-1 cells (Figure 5B and 5C).

Furthermore, CTNNB1 siRNA affected the expression of EMT markers and transcription factors after heat treatment. RT-PCR (Figure 5D) and western blot (Figure 5E) showed that CTNNB1 siRNA restrain the heat-induced EMT. The expression of N-cadherin, vimentin, and Snail was not increased in heated breast cancer cells transfected with si-CTNNB1-1, meanwhile the down regulation of E-cadherin was also diminished.

### ICG001 suppressed metastasis induced by insufficient MWA in SUM-1315 and ZR-75-1 breast cancer cells

**Figure 6:**
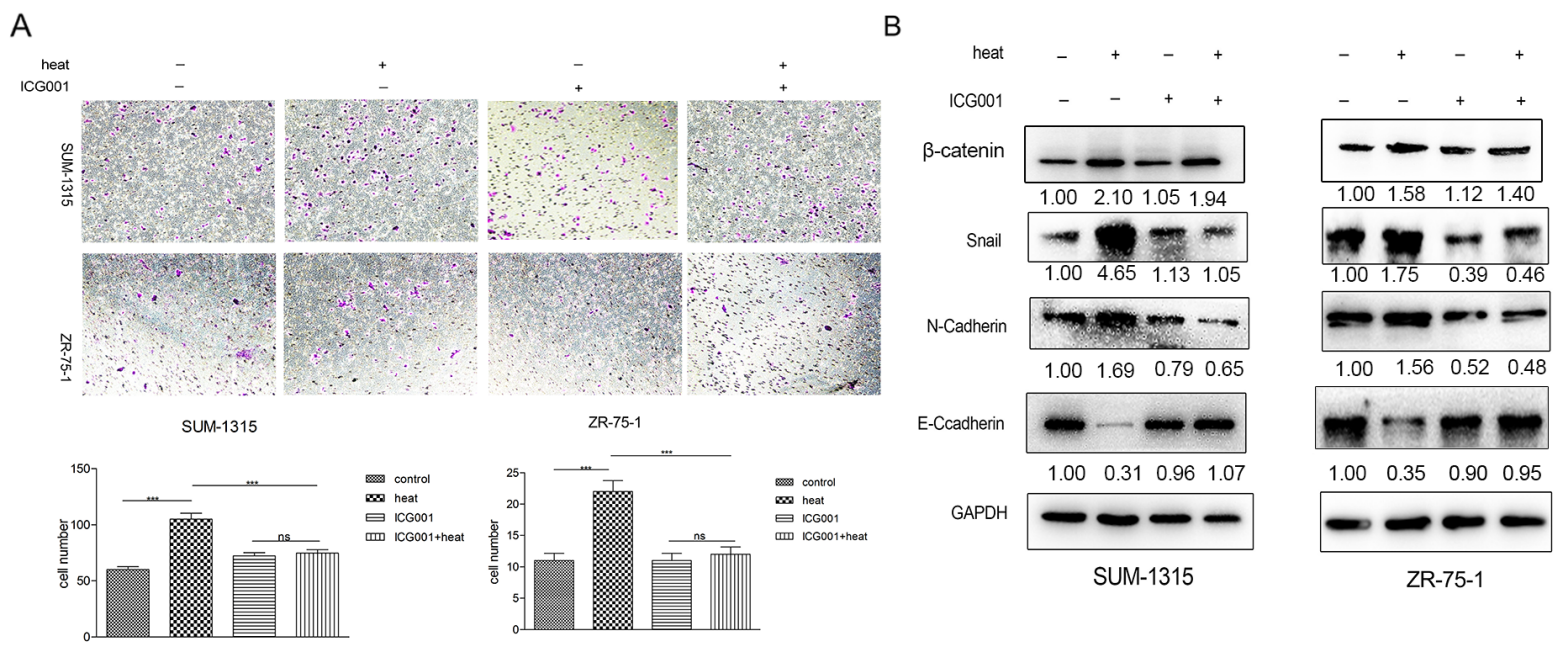
ICG001 suppressed metastasis induced by insufficient MWA in SUM-1315 and ZR-751 breast cancer cells **(A)** Enhanced migration ability was significantly reduced by ICG001 in heated breast cancer cells. **(B)** ICG001 depressed the heat-induced over-expression of EMT markers (N-cadherin, Snail), andpartially recovered the expression of E-cadherin in heat-treated SUM-1315 and ZR-75-1 cells. ^**^p<0.001, ^***^p<0.0001.

After 48h, the heat-treated SUM1315 and ZR-75-1 breast cancer cells were exposed to ICG001 (9μmol/L) for 24h, a special inhibitor of β-catenin pathway. As showed in Figure 6A, ICG001 prevented the spindle morphology and reduced the high migration ability caused by heat treatment in breast cancer cells. Western blot showed that ICG001 depressed the heat-induced over-expression of EMT markers (N-cadherin, Snail), and partially recovered the expression of E-cadherin (Figure 6B).

### ICG001 inhibited insufficient MWA-induced distant metastasis in SUM-1315 nude mice models

**Figure 7:**
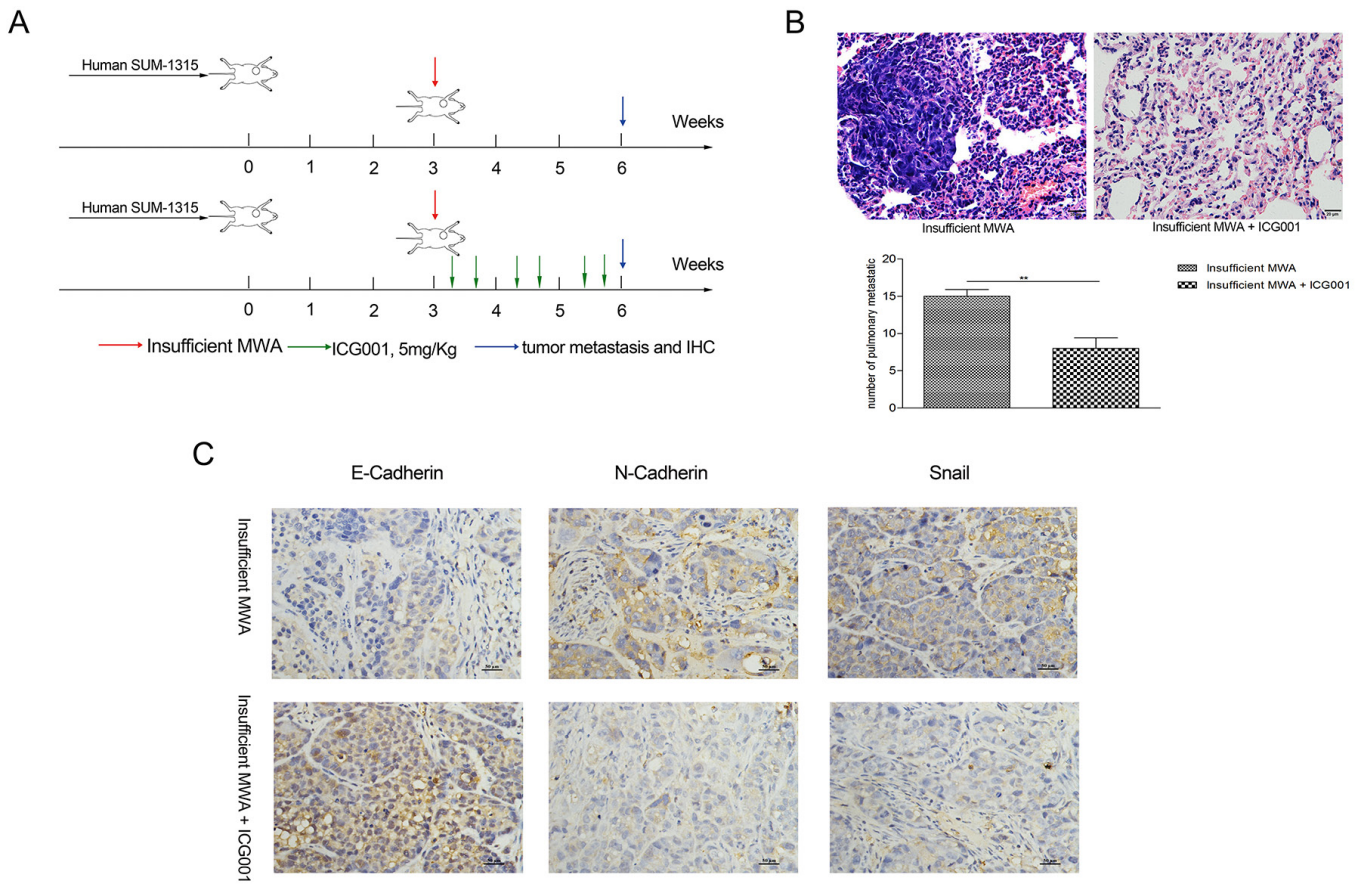
ICG001 inhibited insufficient MWA-induced distant metastasis in SUM-1315 nude mice models **(A)** Experiment design of the *in vivo* test about the effect of ICG001in suppressing insufficient MWA-induced promotion of distant metastasis. **(B)** The increased number of pulmonary metastasis foci caused by insufficient MWA was diminished by ICG001. **(C)** ICG001 normalized the down expression of E-cadherin and reduced the over-expression of N-cadherin and snail in residual tumor tissue after insufficient MWA. ^**^p<0.001.

**Figure 8:**
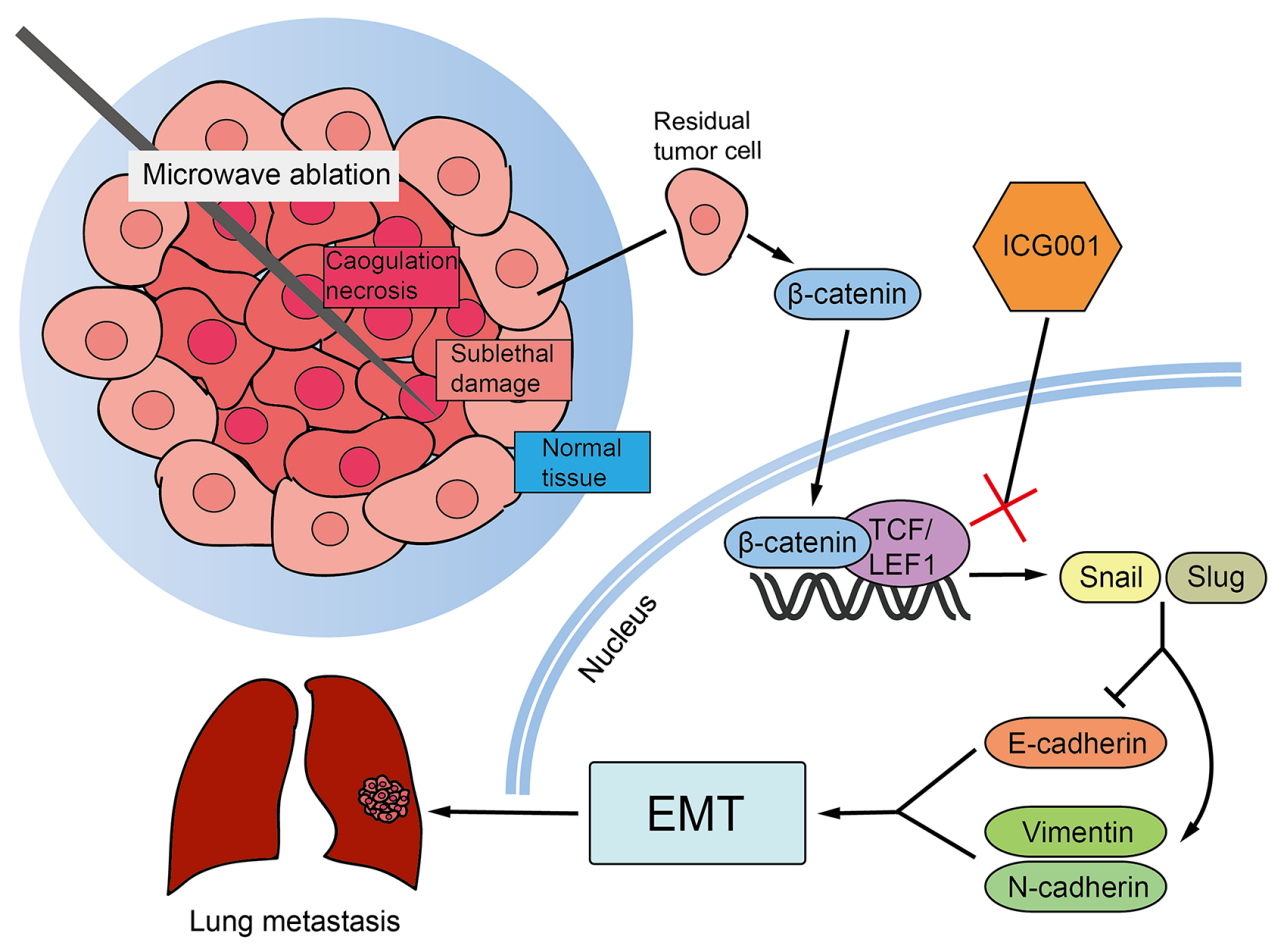
The illustration of β-catenin inhibition on insufficient MWA induced distant metastasis Residual tumors after insufficient MWA were affected by thermal intervention, which influenced EMT-related markers (down-regulated E-cadherin, up-regulated vimentin and N-cadherin) via snail activated by β-catenin, resulting in EMT of residual tumor, while targeting β-catenin pathway by special inhibitor ICG001 neutralized the EMT phenotype and enhanced metastasis caused by insufficient MWA.

To confirm the results *in vivo* (Figure 7A), we examined the effect of ICG001 on residual tumor migration and EMT in SUM-1315 nude model. ICG001 treatment after insufficient MWA significantly decreased the number of metastatic foci in the lung compared with that in insufficient group (8±1.41 *vs* 15±0.11, p<0.05, Figure 7B). IHC presented ICG001 normalized the expression of E-cadherin and reduced the N-cadherin and Snail affected by insufficient MWA (Figure 7C). All these results suggested that insufficient MWA promoted distant metastasis of residual breast cancer, which can be suppressed by β-catenin pathway inhibition (Figure 8).

## DISCUSSION

Residual tumor and local recurrence are the main problems in the treatment of solid tumors with thermal ablation. Several studies have reported residual HCC after RFA shows increased malignant with promoting tumor growth and distant metastasis [11, 22–24]. However, whether the malignancy of residual breast cancer is enhanced after insufficient MWA has remained undetermined. In the present study, for the first time, biological characteristics changes of residual breast cancer after insufficient MWA were reported. *In vitro*, breast cancer cells were exposed to different temperature for 10min to mimic insufficient MWA. With the increasing of temperature, the apoptosis rates increased and the survive rates decreased. The survived cells after heat treatment showed higher migration compared with the parental cells, but the proliferation of survived breast cancer cells did not increase after heat treatment. *In vivo*, we observed that the tumor volume in nude mice breast cancer model was reduced after insufficient MWA, but the pulmonary metastasis increased. The phenomenon observed in our study was different from that in previous study about insufficient RFA of HCC [24]. Different types of tumor and thermal ablation may contribute to these differences.

EMT is an important step in breast cancer metastasis [13, 25, 26]. The tumor cells gain migratory and invasive abilities by EMT, which results in an increased tumor distant metastasis. Our data showed heat treatment induced EMT-like morphology changes of breast cancer cells. Furthermore, down-regulation of E-cadherin, and up-regulation of N-cadherin and vimentin were detected in mRNA levels and protein. In the nude mice breast cancer model, the residual tumor after insufficient MWA also presented the same changes of these EMT markers *in vitro*. These results suggested that insufficient MWA contributed to the EMT of residual tumor, and inhibition of EMT may be a therapeutic strategy for enhanced migration of residual breast cancer.

In order to search for the effective therapy targets, the specific underlying mechanisms should be defined. We found increased β-catenin and EMT transcription factors (Snail and Slug) in heated SUM-1315 and ZR-75-1 cells. Increased β-catenin was also observed in residual tumor after insufficient MWA *in vivo*. After activating of Wnt signal pathway, the quantity of β-catenin in the cytoplasm and nucleus increased [27]. In many cancers [28, 29], the nucleus β-catenin composes a complex with TCF/LEF1 family to inhibit the transcription of CDH1 and induce EMT by directly stimulating Snail and Slug. Additionally, after CTNNB1 siRNA applied to reduce the β-catenin expression, we found the EMT-like morphology induced by heat treatment disappeared, and the differences in migration, EMT markers and transcription factors between heated and non-heated SUM-1315 and ZR-75-1 cells also diminished. Based on the above results, we postulated that residual breast cancer after insufficient MWA presented enhanced metastasis through β-catenin pathway. It provided an important therapy target to prevent the enhanced migration of residual breast cancer after insufficient MWA.

ICG001, a specific inhibitor of Wnt/β-catenin pathway, is a small molecular selectively blocking TCF/β-catenin transcription ina CBP dependent manner [30, 31]. ICG001 and its derivatives have been widely used in preclinical studies [32–34]. However, whether ICG001 can be used to control the enhanced metastasis of residual breast cancer after insufficient MWA has not been reported yet. In our study, ICG001 reduced the migration capacity of heated SUM-1315 and ZR-75-1 cells through inhibition of β-catenin pathway. *In vivo*, ICG001 significantly restrained the lung metastasis, but the tumor volume between the insufficient MWA and the insufficient MWA+ICG group had no difference (data not shown). Our current study appeared to suggest that ICG001 reduced the tumor metastasis mainly by inhibiting EMT in breast cancer. These results also provided favorable basis of using ICG001 to prevent the enhanced metastasis of residual breast cancer after insufficient MWA. However, further studies were needed to clarify the effect of ICG001 on the biological characteristics of breast cancer and the efficiency and security of ICG001 combined with MWA on breast cancer.

Several limitations existed in our study. First, rapid growth of residual breast cancer was not observed, and this still needs to be verified by future studies on different breast cancer cell lines *in vitro* and *in vivo*. Second, we only focused on the effect of heat intervention on the changes of residual tumor in this study, so nude mouse model was applied. However, the effect of immune response induced by MWA [35] to residual tumor cannot be determined. Therefore, biological characteristics changes of residual breast cancer after insufficient MWA in human should be determined in the future.

In summary, our results demonstrated that insufficient MWA promoted EMT of residual breast cancer via β-catenin signal pathway, resulting in enhanced distant metastasis of residual breast cancer. In addition, the effectiveness of ICG001 in suppressing enhanced metastasis of residual breast cancer was preliminarily validated. Obviously, further clinical studies are also needed to confirm these results.

## MATERIALS AND METHODS

### Cell culture and animal

Human breast cancer cell lines ZR-75-1(America Type Culture Center, ATCC) and SUM-1315 (kindly provided by Stephen Ethier, University of Michigan) were cultured in Dulbecco’s Modified Eagle Medium (DMEM, GIBCO, Suzhou, CHINA) containing 10% fetal bovine serum and 1% penicillin-streptomycin in humidified incubator at 37°C with an atmosphere of 5% CO2. The 4-6 weeks old female Balb/c nude mice were purchased from Model Animal Research Center of Nanjing University and maintained under specific pathogen free conditions in Animal Core Facility of Nanjing Medical University. Animal ethics was approved by Institution Animal Care and Use Committee, Nanjing Medical University.

### Heat treatment *in vitro* and construction of residual tumor model after insufficient MWA *in vivo*

Adherence cultured ZR-75-1 and SUM-1315 breast cancer cells were trypsinized and suspensed to DMEM when the cell density reached 60%-70%. 1ml cell suspension (1*10^6^ cells) was placed in 4ml-centrifuge tube and heated in a water bath for 10min. The heat treatment temperature was 42°C, 45°C, 47°C and 50°C, the 37°C as control. The heated cells were seed to 10cm culture dish.

The microwave ablation system (Yigao Microwave Electric Institute, Nanjing, China) was used. The human breast cancer SUM-1315 (2^*^10^6^ suspension in 100μl PBS) were injected on the left side of the second pair of mammary fat pads of nude mice. The tumor diameter reached1.0 to 1.5cm at 3 weeks, and randomly divided into control group (n=4) and insufficient MWA group (n=4). The residual tumor model (insufficient MWA group) was performed as follows: after isoflurane (RWD Life Science, Shenzhen, China) inhalation anesthesia, the mice were fixed and disinfection. A 17-G MWA antenna was inserted into the tumor center along the long axis. Depending on our previously study [35], the MWA was performed at 3W for 3min to ensure the presence of residual tumor.

### CCK8 assay for survive rate and proliferation

3^*^10^3^/well cells were seed to 96-well plate and the cell counting kit-8 (Dojin Laboratories, Kumamoto, Japan) were used to detect the cell survive rate and proliferation according to the instructions. In cell survive rate assay, the SUM-1315 and ZR-75-1 were immediately seed after heat treatment and the test was performed at 48h. At day 3 after heat treatment, the survived cells were inoculated on the 96-well plate and the proliferation test was performed after cultured for 24h, 48h and 72h.

### Flow cytometry for apoptosis

After 48h, the heated cells were trypsinized and washed. 1^*^10^5^ cells were suspended in 1×Binding Buffer and then stained with 5μl Annexin V-FITC and 10μl PI (MULTI SCIENCES, Hangzhou, China), incubated for 5 min, and detected on flow cytometry (Beckman Coulter, Miami, USA). The results were analyzed by FlowJo software (TreeStar, SanCarlos, CA).

### Cell migration assay

The transwell assays (Minicell, Millipore, USA) were used to cell migration assay. After 72h of heat treatment, 200μl serum-free DMEM cell suspension (5^*^10^3^ cells) of survived cells was added into the upper chamber with 8.0μm pore size membrane inserts in 24-well plates. After 24h, the cells migrated to the outside of the membrane were stained with crystal violet (Beyotime, Shanghai, China). The cell number were counted at ×200 magnification, 5 randomly selected regions per well.

### siRNA knockdown for CTNNB1

The CTNNB1 siRNA were synthesized by Shanghai GenePharma Co (Shanghai, China). The SUM-1315 and ZR-75-1 were transfected with CTNNB1 siRNA1, CTNNB1 siRNA2 and CTNNB1 si-mock using Lipofetamine3000 (Invitrogen) according to the instructions. The siRNA sequences are as follows: CTNNB1 siRNA1: 5′-GCAGUUGUGUAAACUUGAUUATT-3′ and 5′-UAA UCAAGUUUACAACUGCTT-3′; CTNNB1 siRNA2: 5′- CCCAAGCUUUAGUAAAUAUTT-3′ and 5′-AUAUU UACUAAAGCUUGGGTT-3′; CTNNB1 si-mock: 5′-UU CUCCGAACGUGUCACGUTT-3′and 5′-ACGUGACAC GUUCGGAGAATT-3′. And the cells were used to further studies after incubating 48h.

### ICG001 treatment *in vitro* and *in vivo*

ICG001 was purchased from MCE (MedChem Express, shanghai, China). After 48h, heat-treated SUM1315 and ZR-75-1 breast cancer cells were exposed to ICG001 (9umol/L) for 24h *in vitro*. *In vivo*, the nude breast cancer models were treated with ICG001 (5mg/Kg, twice a week) by intraperitoneal injection after insufficient MWA.

### Real-time PCR assay

Total mRNA was extracted using the TRIzol reagent (Invitrogen, USA) and reverse transcription was performed using an RT-PCR kit (TaKaRa, Otsu, Japan). The primers sequences used for detecting target genes were showed in Table 1. The procedures of RT-PCR were described elsewhere [36].

**Table 1:** The sequence of primer

Primer		Sequence
GAPDH	Fwd	5′-GAAGGTGAAGGTCGGAGTC-3′
	Rev	5′- GAAGATGGTGATGGGATTTC-3′
Ki-67	Fwd	5′-ACGCCTGGTTACTATCAAAAGG-3′
	Rev	5′- CAGACCCATTTACTTGTGTTGGA-3′
Cyclin D1	Fwd	5′-GCTGCGAAGTGGAAACCATC-3′
	Rev	5′-CCTCCTTCTGCACACATTTGAA-3′
Cyclin E1	Fwd	5′-ACTCAACGTGCAAGCCTCG-3′
	Rev	5′-GCTCAAGAAAGTGCTGATCCC-3′
CDH1	Fwd	5′- CAGCACGTACACAGCCCTAA-3′
	Rev	5′- TGAGGCTTTGGATTCCTCTC-3′
CDH2	Fwd	5′- CAGCACGTACACAGCCCTAA-3′
	Rev	5′- TGAGGCTTTGGATTCCTCTC-3′
Vimentin	Fwd	5′- CAGATGCGTGAAATGGAAGA-3′
	Rev	5′- CTCAATGTCAAGGGCCATCT-3′
Snail	Fwd	5′-CCTCCACGAGGTGTGACTAACT-3′
	Rev	5′-CCGACAAGTGACAGCCATTA -3′
Slug	Fwd	5′-CGCAATCAATGTTTACTCGAAC-3′
	Rev	5′- TCTCAATCTAGCCATCAGCAAA-3′
Twist	Fwd	5′-CATCGACTTCCTCTACCAGGTC-3′
	Rev	5′-CCTTCTCTGGAAACAATGACATC-3′
Zeb1	Fwd	5′- GTCCCACACGACCACAGAT-3′
	Rev	5′- ATGGGAGACACCAAACCA-3′
CTNNB1	Fwd	5′- CCTATGCAGGGGTGGTCAAC-3′
	Rev	5′- CGACCTGGAAAACGCCATCA-3

### Western blot assay

The procedure of western blot has been described in previous studies [37]. The primary antibodies to E-cadherin, N-cadherin, Snail and Slug were purchased from Cell Signaling Technology (CST, Boston, MA, USA). The anti-vimentin and anti-β-catenin were obtained from Abcam (Cambridge, UK). The antibody of GAPDH was provided by Beyotime (Shanghai, China).

### Immunohistochemistry (IHC)

The tumor tissues were embedded in paraffin and cut into 4μm thickness sections. The sections were stained with E-cadherin, N-cadherin, Snail and β-catenin, then incubated with the appropriate HRP conjugated secondary antibody followed by standard streptavidinbiotin method by using the 3,3′-Diaminobenzidine (DAB) substrate.

### Statistics

All the data were statistical analyzed by SPSS version 22 (SPSS, Chicago, USA). Student’s *t*-test or one-way ANOVA was used to perform the statistical analysis, p<0.05 was considered as statistically significant.

## SUPPLEMENTARY MATERIALS FIGURE



## Abbreviations

MWA: microwave ablation
RFA: radiofrequency ablation
HCC: hepatocelluar carcinoma
EMT: epithelial-mesenchymal transition
TGF-β: transform growth factor β;
