# Supplementary material for: Insufficient microwave ablation-induced promotion of distant metastasis is suppressed by β-catenin pathway inhibition in breast cancer
Source: Oncotarget. 2017 Dec 1;8(70):115089–101. doi: 10.18632/oncotarget.22859 (PMC5777756; doi:10.18632/oncotarget.22859)
Supplement: Supplementary file 1 [file oncotarget-08-115089-s001.pdf]

Insufficient microwave ablation-induced promotion of distant metastasis is suppressed by β-catenin pathway inhibition in breast cancer

SUPPLEMENTARY MATERIALS

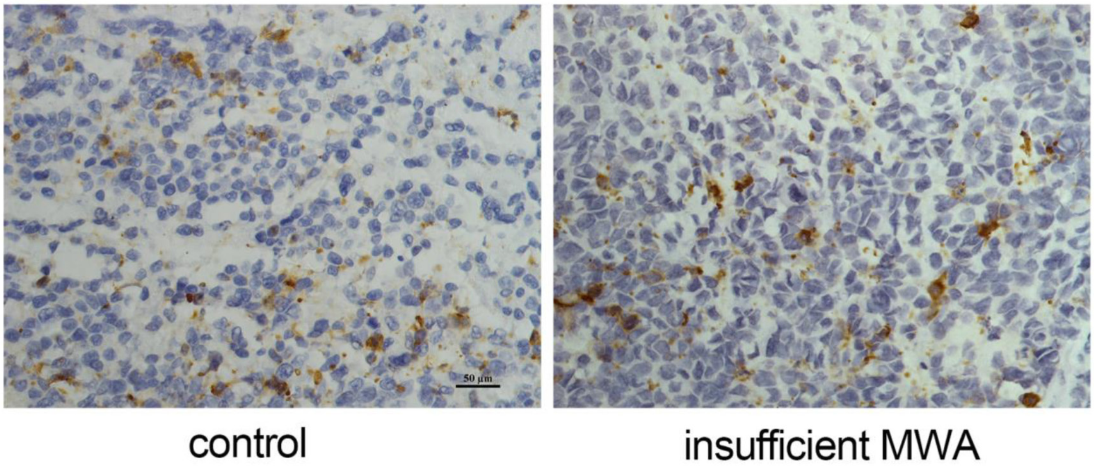

Supplementary Figure 1: The apoptosis marker caspase-3 had no difference between these two groups. The caspase-3 was detected by IHC at 21 days after insufficient MWA.

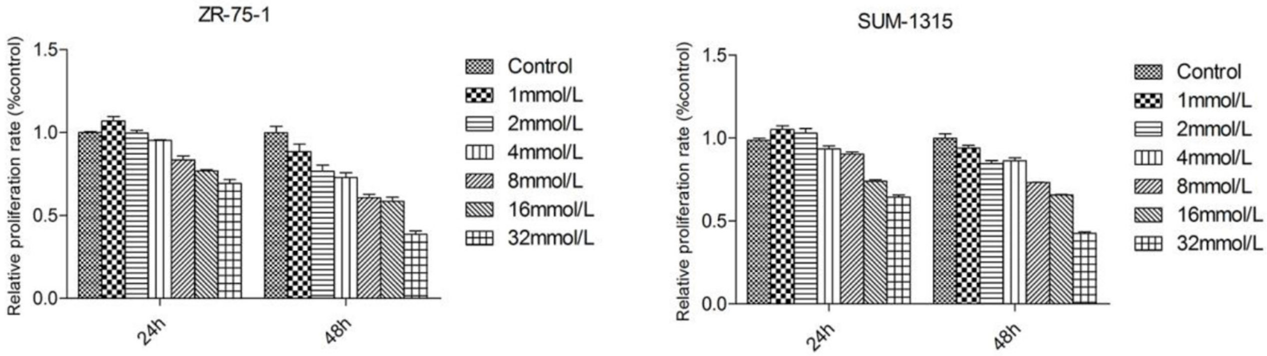

Supplementary Figure 2: ICG001 inhibit the cell growth of breast cancer ZR-75-1 and SUM-1315. The breast cancer cells were treated with different concentration of ICG001, and the cell proliferation was detected by CCK8 assay at 24h and 48h. The results showed that ICG001 could inhibit the cell growth with a time and dose-dependence.

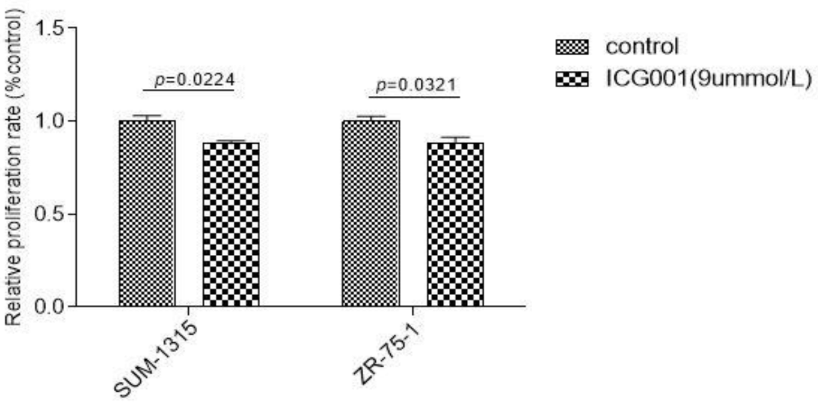

Supplementary Figure 3: ICG001 (9ummol/L) had a weak ability to inhibit breast cancer cell growth. The breast cancer cells were treated with ICG001 (9ummol/L) for 24h, and the cell proliferation rate of the treated SUM-1315 and ZR-75-1 was 87.84% and 84.66%, respectively.
